# Surveillance and follow up outcomes of myocarditis after mRNA COVID-19 vaccination in Australia

**DOI:** 10.1038/s41541-025-01206-w

**Published:** 2025-07-16

**Authors:** Lucy Deng, Amanda Van Eldik, Megan O’Moore, Jim Buttery, Abigail Cheung, Nicholas Cox, Carla Drake-Brockman, Nathan Dwyer, Paul Effler, Michael Gold, Pravin Hissaria, Andrew Kelly, Sarah Khanlari, Claire Larter, Shannon Melody, Michael Nissen, Rajesh Puranik, James Rankin, Sally Singleton, Liza Thomas, Sudhir Wahi, Gavin Wheaton, Dominica Zentner, Kristine Macartney, Clara K. Chow, Nicholas Wood

**Affiliations:** 1 National Centre for Immunisation Research and Surveillance, Children’s Hospital at Westmead, Westmead, NSW Australia; 2 The University of Sydney Children’s Hospital Westmead Clinical School, Westmead, NSW Australia; 3 Therapeutic Goods Administration, Department of Health, Disability and Ageing, Canberra, ACT Australia; 4 Murdoch Children’s Research Institute, Parkville, Victoria Australia; 5 Department of Paediatrics, University of Melbourne, Parkville, Victoria Australia; 6 Women’s and Children’s Health Network, Adelaide, South Australia Australia; 7 Western Health, Victoria, Australia; 8 Royal Melbourne Hospital Clinical School, Department of Medicine, University of Melbourne, Melbourne, Victoria Australia; 9 Perth Children’s Hospital, Nedlands, Western Australia Australia; 10 Royal Hobart Hospital, Hobart, Tasmania Australia; 11 Western Australia Department of Health, Perth, Western Australia Australia; 12 School of Medicine, Adelaide University, Adelaide, South Australia Australia; 13 South Australia Pathology, Adelaide, South Australia Australia; 14 New South Wales Ministry of Health, St Leonards, NSW Australia; 15 Public Health Services, Department of Health Tasmania, Hobart, Tasmania Australia; 16 Queensland Adult Specialist Immunisation Service, The Prince Charles Hospital, Chermside, Queensland Australia; 17 Royal Prince Alfred Hospital, Camperdown, NSW Australia; 18 Fiona Stanley Hospital, Murdoch, Western Australia Australia; 19 Australian Capital Territory Health Directorate, Canberra, ACT Australia; 20 The University of Sydney’s Westmead Clinical School, Westmead, NSW Australia; 21 Westmead Hospital, Westmead, NSW Australia; 22 Princess Alexandra Hospital, Brisbane, Queensland Australia; 23 Royal Melbourne Hospital, Parkville, Victoria Australia; 24 Westmead Applied Research Centre, Faculty of Medicine and Health, The University of Sydney, Westmead, NSW Australia

**Keywords:** RNA vaccines, Outcomes research

## Abstract

Clinical progression and medium-long term morbidity from myocarditis following mRNA COVID-19 vaccinations remains an important but undefined public health concern. We conducted prospective follow-up of individuals with either confirmed or probable myocarditis following monovalent Pfizer-BioNTech BNT162b2 or Moderna mRNA-1273 vaccination between 21 April 2021 and 5 July 2022 in Australia. Of 256 individuals who consented to follow up, mostly males following a second dose, 60% (133/221) had ongoing symptoms at 3-6 months and 35% (81/231) at 12-18 months. Self-reported ongoing exercise restrictions, medication requirements, and hospital re-presentations were associated with ongoing symptoms, as was a lower self-reported health status and quality of life. Clinical severity remained mild, with low hospitalisation rates and no deaths in the follow-up period and health-related quality of life improved over time. These findings support ongoing use of mRNA COVID-19 vaccines in at-risk individuals to prevent disease caused by SARS-CoV-2 infection.

## Introduction

Myocarditis, an inflammatory condition of the myocardium, has been associated with COVID-19 vaccination, typically within 7 days of vaccination. The highest attributable risk was seen in males aged 12-29 years, and occurred following a second dose of a mRNA COVID-19 vaccine^1–5^. Myocarditis from other causes, including *SARS*-*CoV*-*2* infection, other viral infections, drugs and those with no identifiable aetiology, have a highly variable clinical progression with prognosis ranging from spontaneous resolution without treatment, to dilated cardiomyopathy and cardiac failure requiring heart transplantation.

Early case series of myocarditis following mRNA COVID-19 vaccination suggested most individuals typically have a mild clinical presentation and self-limiting course, with symptom resolution at or soon after initial discharge^6–11^. A small minority of individuals had severe disease at diagnosis^12,13^. At follow-up, one surveillance cohort study of over 500 individuals aged 12-29 years with myocarditis following mRNA COVID-19 vaccination meeting the United States Centre for Disease Control and Prevention (CDC) case definition criteria^14^ found most patients remained clinically well 90 days following diagnosis^15^. Only 2% of patients had a further hospital admission and 80% were considered fully recovered by their healthcare provider. However, half the cohort self-reported symptoms at 3-6 months, including some who were considered fully recovered by their healthcare worker. Of these, 30% reported pain/discomfort and 46% reported anxiety/depression symptoms. Similarly, Shenton et al.^16^ found half of 182 cases of myocarditis following mRNA COVID-19 vaccination in Victoria, Australia reported ongoing symptoms 6 months following their initial myocarditis diagnosis. In the longest follow-up study to date using health datasets only, individuals with myocarditis following mRNA COVID-19 vaccination had lower frequency of re-hospitalisation for any cardiovascular event than individuals with myocarditis of other causes at 18 months following diagnosis^17^.

Little is known about the clinical progression of myocarditis beyond symptoms in the initial 6 months and hospitalisation rates at 18 months. It is key to understand if myocarditis following mRNA COVID-19 vaccination is associated with longer term morbidity as mRNA COVID-19 vaccines continue to be used. In this national prospective Australian cohort study, we aimed to systematically assess the clinical status and health-related quality of life by following up individuals with myocarditis associated with mRNA COVID-19 vaccination for 18 months.

## Results

**Fig. 1:**
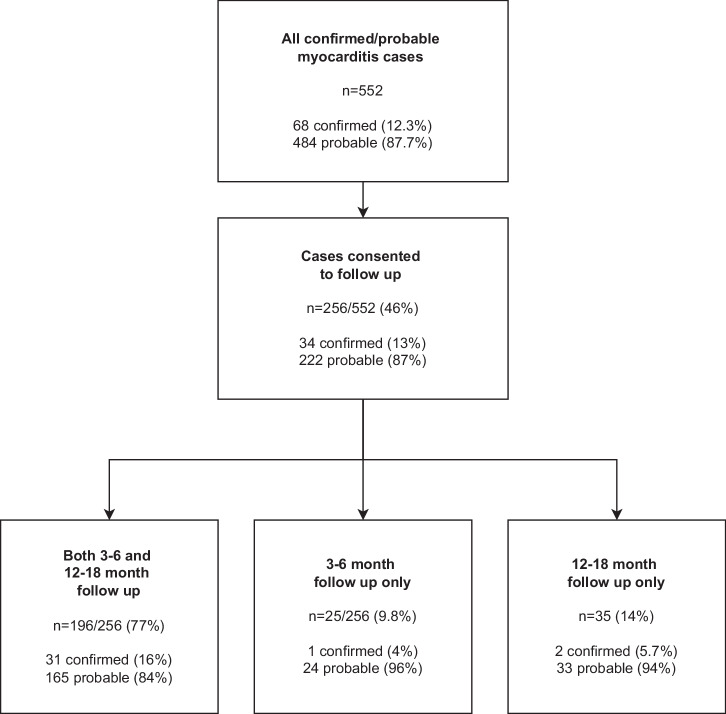
Study population and follow up recruitment.

There were 552 cases of myocarditis (68 (12%) confirmed and 484 (88%) probable cases as classified by the U.S CDC case definition (Supplementary Table 1)^14^ identified in Adverse events following immunisation (AEFI) reports to Australia’s national regulatory agency, the Therapeutic Goods Administration (TGA) between February 2021 and July 2022 (Fig. 1).

### Demographic and clinical data at presentation (*n* = 552)

Of the 552 cases, 487 (88%) were following Pfizer–BioNTech BNT162b2 and 65 (12%) following Moderna mRNA-1273, equating to a rate of 1.2 cases per 100,000 Pfizer–BioNTech BNT162b2 doses and 1.4 cases per 100,000 Moderna mRNA-1273 doses administered. Most myocarditis cases occurred after administration of dose 2 (70%), with fewer cases occurring after dose 1 (20%) and dose 3 (9.4%). The median age was 22 years [interquartile range IQR 17–33, range 5–76 years]. There were three times as many male cases as female cases (76% male vs 24% female) (Table 1). There was no difference in age, sex or Indigenous status when comparing confirmed with probable cases (Supplementary Table 2)

**Table 1:** Demographics, past medical history, clinical and diagnostic findings of initial presentation in 552 confirmed and probable myocarditis cases following mRNA COVID-19 vaccination presenting between 21 April 2021 and 5 July 2022, comparing cases who participated in any follow up and those who did not

	All cases *N* = 552	Cases with no follow up *N* = 296	Cases with follow up* *N* = 256	*P*
**Case classification**				
Confirmed	68 (12%)	34 (11%)	34 (13%)	0.5
Probable	484 (88%)	262 (89%)	222 (87%)	
**Vaccine brand**				
Pfizer–BioNTech BNT162b2	487 (88%)	263 (89%)	224 (88%)	0.6
Moderna mRNA-1273	65 (12%)	33 (11%)	32 (12%)	
**Vaccine dose**				
Dose 1	111 (20%)	63 (21%)	48 (19%)	0.006
Dose 2	389 (70%)	216 (73%)	173 (68%)	
Dose 3	52 (9.4%)	17 (5.7%)	35 (14%)	
**Age (years)**				
Median [IQR]	22 [17–33]	20 [17–29]	24 [17–37]	<0.001
0–11	6 (1.1%)	1 (0.3%)	5 (2.0%)	
12–14	70 (13%)	44 (15%)	26 (10%)	
15–19	165 (30%)	105 (35%)	60 (23%)	
20–24	90 (16%)	49 (17%)	41 (16%)	
25–29	49 (8.9%)	29 (9.8%)	20 (7.8%)	
30–39	145 (26%)	62 (21%)	83 (32%)	
≥40	27 (4.9%)	6 (2.0%)	21 (8.2%)	
**Sex**				
Male	418 (76%)	229 (77%)	189 (74%)	0.2
Female	132 (24%)	65 (22%)	67 (26%)	
Another term	2 (0.4%)	2 (0.7%)	0 (0%)	
**Aboriginal and/or Torres Strait Islander**	16 (2.9%)	8 (2.7%)	8 (3.1%)	0.8
**Past medical history**				
Cardiovascular condition^a^	24 (4.3%)	9 (3.0%)	15 (5.9%)	0.1
Myocarditis	9 (1.6%)	4 (1.4%)	5 (2.0%)	0.7
Pericarditis	7 (1.3%)	2 (0.7%)	5 (2.0%)	0.3
Arrhythmia	6 (1.1%)	1 (0.3%)	5 (2.0%)	0.1
Congenital heart disease	4 (0.7%)	2 (0.7%)	2 (0.8%)	0.9
Autoimmune/systemic inflammatory condition^b^	19 (3.4%)	5 (1.7%)	14 (5.5%)	0.02
Genetic/chromosomal condition	10 (1.8%)	3 (1.0%)	7 (2.7%)	0.1
**Family history of cardiac conditions**^**c**^	29 (5.3%)	5 (1.7%)	24 (9.4%)	<0.001
**Time to symptom onset**				
0–7 days	465 (84%)	252 (85%)	213 (83%)	0.6
8–10 days	22 (4.0%)	12 (4.1%)	10 (3.9%)	
≥ 11 days	65 (12%)	32 (11%)	33 (13%)	
**Presenting symptoms**				
Cardiovascular				
Chest pain, pressure, discomfort	530 (96%)	283 (96%)	247 (96%)	0.6
Heart palpitations	122 (22%)	57 (19%)	65 (25%)	0.08
Diaphoresis	52 (9.4%)	23 (7.8%)	29 (11%)	0.2
Syncope	12 (2.2%)	2 (0.7%)	10 (3.9%)	0.01
Dizziness	49 (8.9%)	16 (5.4%)	33 (13%)	0.002
Respiratory				
Shortness of breath	190 (34%)	94 (32%)	96 (38%)	0.2
Cough	31 (5.6%)	16 (5.4%)	15 (5.9%)	0.8
Pleuritic chest pain	79 (14%)	41 (14%)	38 (15%)	0.7
Gastrointestinal				
Abdominal pain	11 (2.0%)	4 (1.4%)	7 (2.7%)	0.2
Nausea/vomiting	70 (13%)	28 (9.5%)	42 (16%)	0.01
Diarrhoea	5 (0.9%)	1 (0.3%)	4 (1.6%)	0.2
Loss of appetite	7 (1.3%)	1 (0.3%)	6 (2.3%)	0.05
Systemic				
Fever	73 (13%)	29 (9.8%)	44 (17%)	0.01
Headache	57 (10%)	20 (6.8%)	37 (14%)	0.003
Lethargy	103 (19%)	39 (13%)	64 (25%)	0.003
**Investigations**				
Abnormal ECG	354/522 (64%)	189/278 (68%)	165/244 (68%)	
Abnormal echocardiogram	180/487 (33%)	89/260 (34%)	91/227 (40%)	
Abnormal cMRI^d^	128/149 (86%)	73/80 (91%)	55/69 (80%)	
Elevated troponin	524/552 (95%)	278/278 (100%)	246/274 (90%)	
Abnormal biopsy^e^	2/3 (67%)	1/2 (50%)	1/1 (100%)	
**Management details**				
Treated at GP	15 (2.7%)	11 (3.7%)	4 (1.6%)	0.03
Treated in ED	74 (14%)	42 (15%)	32 (13%)	
Hospitalised, no ICU admission	443 (82%)	238 (84%)	205 (81%)	
Hospitalised, with ICU admission	20 (3.7%)	5 (1.8%)	15 (6.0%)	
**Intervention**				
Non-invasive cardiorespiratory support	10 (1.8%)	3 (3.7%)	7 (2.7%)	0.03
Intubation and ventilation	2 (0.4%)	1 (0.3%)	1 (0.4%)	
ECMO	1 (0.2%)	1 (0.3%)	0 (0%)	
Procedures/surgery	12 (3.0%)	3 (1.5%)	9 (4.5%)	
**Discharge medications**				
Anti-inflammatories^f^	485 (88%)	259 (88%)	219 (86%)	
Corticosteroids^g^	18 (3.3%)	8 (2.7%)	10 (3.9%)	0.4
Anti-arrhythmic	1 (0.2%)	0 (0%)	1 (0.4%)	0.5
Anticoagulation	33 (6%)	16 (5.4%)	17 (6.6%)	0.5
Diuretics	3 (0.5%)	1 (0.3%)	2 (0.8%)	0.6
Other cardiac medications^h^	81 (15%)	36 (12%)	45 (18%)	0.07
**Discharge outcomes**				
Ongoing symptoms	147 (27%)	24 (8.1%)	123 (48%)	<0.001
Placed on physical activity restrictions	291 (53%)	123 (42%)	168 (66%)	<0.001

^*^Cases with follow up includes those who consented to the study and responded to the 3-6 month follow-up and/or 12-18 month follow-up.

cMRI cardiac magnetic resonance imaging, ECG electrocardiogram, ECMO extra corporeal. membrane oxygenation, ED emergency department, GP general practice, ICU intensive care unit, IQR interquartile range.

^a^Cardiovascular condition includes myocarditis, pericarditis, arrhythmia, congenital heart disease.

^b^Autoimmune/systemic inflammatory condition includes rheumatoid arthritis, scleroderma, systemic lupus erythematosus, Sjogren’s syndrome, Kawasaki disease, inflammatory bowel disease.

^c^Family history of cardiac conditions includes sudden cardiac death, cardiomyopathy, permanent pacemaker, implantable cardioverter defibrillator device.

^d^Abnormal cMRI includes any cMRI findings consistent with myocarditis.

^e^Abnormal biopsy includes histopathology confirmation of myocarditis.

^f^Anti-inflammatory medications include aspirin, ibuprofen, naproxen and colchicine.

^g^ Corticosteroids include prednisone, dexamethasone and methylprednisone

^h^ Other cardiac medications include angiotensin-converting enzyme inhibitors, angiotensin receptor blockers and beta blocker

### Acute clinical course

Most cases presented within 7 days (84%) of their COVID-19 vaccine (median time to onset: 3 days [IQR 1–4]), with no difference in timing between confirmed and probable cases. The most common presenting symptom was chest pain/pressure/discomfort (96%) followed by shortness of breath (34%) (Table 1). Confirmed cases were more likely to have pleuritic chest pain (28% vs 12%) and fever (22% vs 12%) compared to probable cases (Supplementary Table 2).

Most individuals were hospitalised (86%) with a median length of hospital stay of 2 days [IQR 2-3]. Twenty cases (3.7%) required intensive care unit (ICU) admission, with two individuals requiring intubation and mechanical ventilation and one requiring extracorporeal membrane oxygenation. Twelve individuals (3%) (10 males (83%) and 2 females (17%), median age 41 years [IQR: 25-49]) reported having procedures or surgery during their admission. There were nine (75%) cardiac procedures; eight coronary angiography/catheterisation and one pericardiocentesis. There were three non-cardiac procedures; a cholecystectomy, an arthroscopy and a muscle biopsy for investigation of an alternate aetiology. All hospitalised individuals were discharged home and there were no deaths.

Ongoing symptoms at time of discharge were reported in 27% of cases, mostly with ongoing chest pain. Most individuals (88%) were discharged on anti-inflammatory medication, 53% were advised to restrict physical activity. Confirmed cases had a higher rate of corticosteroid and non-antiarrhythmic medication requirements on discharge and were more likely to be advised of physical restrictions on discharge (Supplementary Table 2).

### Diagnostic findings

Cardiac magnetic resonance imaging (cMRI) was performed at initial diagnosis for 149 individuals, of which 128 (83%) were abnormal (57/57 (100%) of confirmed and 71/92 (77%) of probable cases). Of those who had cMRIs, the median age was 22 years, and most cases were male (83%) and following dose 2 (71%).

There were three cases with histopathology performed on cardiac biopsies as part of initial assessment, all in males aged 34–53 years and following dose 2 Pfizer–BioNTech BNT162b2. All had prolonged hospitalisations (13–16 days) and two required ICU admission. Two showed lymphocytic infiltrates and evidence of myocyte necrosis and degeneration consistent with lymphocytic myocarditis while the other found no evidence of myocarditis or pericarditis.

### Follow up cohort (*n* = 256)

Of the 552 myocarditis cases, 256 (46%) consented to follow up and completed at least one follow up: 221 (86%) completed the 3–6 month follow up survey (median time to follow up 109 days [IQR 96–140]), 231 (90%) completed the 12–18 month follow up survey (median time to follow up 402 days [IQR 385–444]), and 196 (77%) completed both (Fig. 1).

Follow-up cases were aged 7–76 years with the median age higher in those who consented for follow-up than those who did not (24 [IQR 17–37] years vs 20 [IQR 17–29] years, *P* < 0.05). There was a higher proportion of cases with a family history of cardiac conditions of interest (sudden cardiac death, cardiomyopathy, permanent pacemaker and/or implantable cardioverter defibrillator or cardiac adverse event to any previous vaccine), but a lower proportion of past medical conditions in the follow-up group compared to the no follow-up group. There was no sex difference or difference in proportion of Aboriginal and Torres Strait Islanders participating in follow-up. Individuals who had follow-up had higher rates of syncope, dizziness, headache, and lethargy compared to individuals who did not have follow-up. They also had more severe disease with a higher proportion of individuals requiring ICU admission (6% vs 1.8%) and reporting ongoing symptoms (48% vs 8.1%) and exercise restrictions (66% vs 42%) at discharge (Table 1).

### Clinical progression

**Fig. 2:**
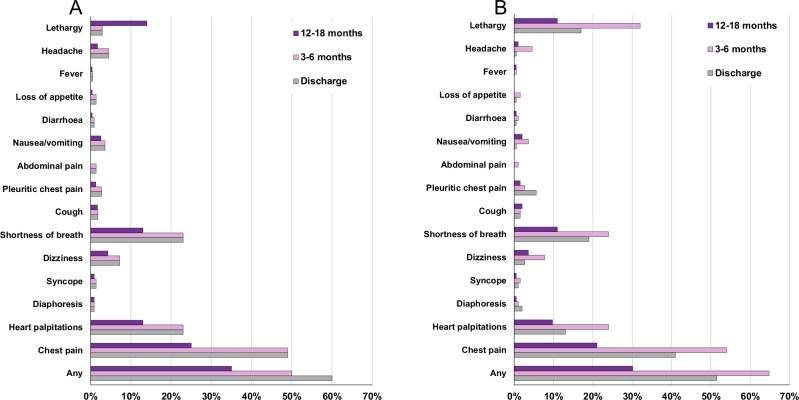
Ongoing symptoms in myocarditis following a COVID-19 mRNA vaccine. **A** Cases who completed any follow-up: discharge (*n* = 256), 3-6 months (*n* = 221) and 12-18months (*n* = 231). **B** Cases who completed all follow-up timepoints (*n* = 196).

The proportion of individuals who had follow-up and reported ongoing symptoms was 48% (123/256) at discharge, 60% (133/221) at 3–6 months, and 35% (81/231) at 12–18 months (Table 2). Chest pain, heart palpitations, and shortness of breath were the most reported symptoms (Table 2, Fig. 2a). While there was no difference in the proportion of males and females reporting ongoing symptoms at discharge or at 3–6 months, there was a significantly higher proportion of females reporting ongoing symptoms at 12–18 months compared to males. There was a higher proportion of females reporting palpitations at all time points, lethargy/fatigue at both follow-up time points, and shortness of breath at 12–18 months compared to males. Ongoing symptoms at 12–18 months was also associated with older age (Table 3).

**Table 2:** Clinical features in 256 cases of myocarditis following a COVID-19 mRNA vaccine who participated in follow-up, at discharge following initial diagnosis, 3–6 months and 12–18 months following initial diagnosis and by sex

	Discharge			3–6 month follow up			12–18 month follow up		
	All	Male	Female	All	Male	Female	All	Male	Female
	*N* = 256	*N* = 189	*N* = 67	*N* = 221	*N* = 167	*N* = 54	*N* = 231	*N* = 170	*N* = 61
**Any ongoing symptoms**	123 (48%)	93 (49%)	30 (45%)	133 (60%)	97 (58%)	36 (67%)	81 (35%)	50 (29%)*	31 (51%)*
**Cardiovascular symptoms**									
Chest pain, pressure or discomfort	99 (39%)	74 (39%)	25 (37%)	109 (49%)	79 (47%)	30 (56%)	58 (25%)	41 (24%)	17 (28%)
Palpitations	28 (11%)	16 (8.5%)*	12 (18%)*	50 (23%)	29 (17%)	21 (39%)	30 (13%)	15 (8.8%)*	15 (25%)*
Diaphoresis	2 (0.8%)	1 (0.5%)	1 (1.5%)	2 (0.9%)	2 (1.2%)	0 (0%)	2 (0.9%)	1 (0.6%)	1 (1.6%)
Syncope	2 (0.8%)	1 (0.5%)	1 (1.5%)	3 (1.4%)	2 (1.2%)	1 (1.9%)	2 (0.9%)	0 (0%)	2 (3.3%)
Dizziness	8 (3.1%)	5 (2.6%)	3 (4.5%)	16 (7.2%)	8 (4.8%)	8 (15%)	10 (4.3%)	8 (4.7%)	2 (3.3%)
**Respiratory symptoms**									
Shortness of breath	48 (19%)	36 (19%)	12 (18%)	50 (23%)	37 (22%)	13 (24%)	31 (13%)	17 (10%)*	14 (23%)*
Cough	4 (1.6%)	2 (1.1%)	2 (3.0%)	4 (1.8%)	0 (0%)	4 (7.4%)	4 (1.7%)	3 (1.8%)	1 (1.6%)
Pleuritic chest pain	11 (4.3%)	7 (3.7%)	4 (6.0%)	6 (2.7%)	4 (2.4%)	2 (3.7%)	3 (1.3%)	1 (0.6%)	2 (3.3%)
**Gastrointestinal symptoms**									
Abdominal pain	0 (0%)	0 (0%)	0 (0%)	3 (1.4%)	3 (1.8%)	0 (0%)	0 (0%)	0 (0%)	0 (0%)
Nausea/vomiting	1 (0.4%)	0 (0%)	1 (1.5%)	8 (3.6%)	4 (2.4%)	4 (7.4%)	6 (2.6%)	3 (1.8%)	3 (4.9%)
Diarrhoea	1 (0.4%)	1 (0.5%)	0 (0%)	2 (0.9%)	2 (1.2%)	0 (0%)	1 (0.4%)	1 (0.6%)	0 (0%)
Loss of appetite	1 (0.4%)	0 (0%)	1 (1.5%)	3 (1.4%)	2 (1.2%)	1 (1.9%)	1 (0.4%)	0 (0%)	1 (1.6%)
**Systemic symptoms**									
Fever	0 (0%)	0 (0%)	0 (0%)	1 (0.5%)	0 (0%)	1 (1.9%)	1 (0.4%)	1 (0.6%)	0 (0%)
Headache	1 (0.4%)	0 (0%)	1 (1.5%	10 (4.5%)	6 (3.6%)	4 (7.4%)	4 (1.7%)	2 (1.2%)	2 (3.3%)
Lethargy/fatigue	39 (15%)	31 (16%)	8 (12%)	65 (29%)	42 (25%)*	23 (43%)*	32 (14%)	17 (10%)*	15 (25%)*
**On exercise/physical activity restrictions**	168 (66%)	136 (72%)*	32 (48%)*	84 (38%)	67 (40%)	17 (31%)	19 (8%)	12 (7%)	7 (12%)
**Hospital re-presentation relating to their myocarditis**				60 (27%)	38 (23%)*	22 (41%)*	47 (20%)	29 (17%)*	18 (30%)*
ED presentation				50 (23%)	30 (18%)	20 (37%)	41 (18%)	24 (14%)	17 (28%)
Hospitalisation				19 (8.6%)	11 (6.6%)	8 (15%)	13 (5.6%)	9 (5.3%)	4 (6.6%)
ICU admission				1 (0.5%)	0 (0%)	1 (1.9%)	3 (1.3%)	2 (1.2%)	1 (1.6%)
**Medications**	235 (92%)	177 (94%)	58 (87%)	76 (34%)	50 (30%)*	26 (48%)*	31 (13%)	18 (11%)*	13 (21%)*
Anti-inflammatories^a^	223 (87%)	171 (90%)	52 (78%)	57 (26%)	40 (24%)	17 (31%)	20 (9%)	13 (7.6%)	7 (11%)
Corticosteroids^b^	10 (3.9%)	6 (3.2%)	4 (6.0%)	2 (0.9%)	2 (1.2%)	0 (0%)	2 (0.9%)	1 (0.6%)	1 (1.6%)
Anti-arrhythmic	1 (0.4%)	1 (0.5%)	0 (0%)	1 (0.5%)	1 (0.6%)	0 (0%)	0 (0%)	0 (0%)	0 (0%)
Anticoagulation	17 (6.6%)	8 (4.2%)	9 (13%)	3 (1.4%)	0 (0%)	3 (5.6%)	0 (0%)	0 (0%)	0 (0%)
Diuretics	2 (0.8%)	1 (0.5%)	1 (1.5%)	0 (0%)	0 (0%)	0 (0%)	1 (0.4%)	1 (0.6%)	0 (0%)
Other cardiac medications^c^	45 (18%)	32 (17%)	13 (19%)	17 (8%)	11 (6.6%)	6 (11%)	16 (7%)	10 (5.9%)	6 (9.8%)
**Another COVID-19 vaccination**				37 (16.7%)	23 (13.8%)	14 (25.9%)	53 (22.9%)	32 (18.8%)	21 (34.4%)
**Another adverse event following immunisation**				13^d^	7	6	18^e^	10	8

^*^P < 0.05.

ED=emergency department, ICU=intensive care unit.

^a^Anti-inflammatory medications include aspirin, ibuprofen, naproxen and colchicine.

^b^Corticosteroids include prednisone, dexamethasone and methylprednisone.

^c^Other cardiac medications include ACE inhibitors, angiotensin receptor blockers and beta blockers.

^d^Includes 3 cases of self-resolving cardiorespiratory symptoms with no diagnoses of myocarditis with no hospitalisation.

^e^Includes 8 cases of self-resolving cardiorespiratory symptoms with no diagnoses of myocarditis with no hospitalisation.

**Table 3:** Clinical progression and self-assessment of health-related quality of life on EuroQol 5-dimension, 5-level (EQ-5D-5L) and overall health on EuroQol Visual Analogue Scale (EQ-VAS) among myocarditis cases following mRNA COVID-19 vaccination at 3–6 and 12–18 months, by ongoing symptoms reported at follow up

	3–6 month follow up					12–18 month follow up				
	All	No ongoing symptoms	Ongoing symptoms	*P*	aOR (95% CI)^1^	All	No ongoing symptoms	Ongoing symptoms	*P*	aOR (95% CI)^1^
**Clinical progression**^**2**^	***N*** = **221**	***N*** = **88**	***N*** = **133**			***N*** = **231**	***N*** = **150**	***N*** = **81**		
Medication	76 (34%)	12 (14%)	64 (48%)	<0.001	3.52 (1.62–7.67)	31 (13%)	5 (3.3%)	26 (32%)	<0.001	7.61 (2.57-22.57)
Exercise/Physical activity restriction	84 (38%)	11 (13%)	73 (55%)	<0.001	6.13 (2.84–13.24)	19 (8.2%)	1 (0.7%)	18 (22.2%)	<0.001	33.84 (4.11-278.16)
Hospital representation	60 (27%)	12 (14%)	48 (36%)	<0.001	2.01 (0.89–4.51)	47 (20%)	23 (15%)	24 (30%)	0.01	2.17 (1.00-4.72)
**Health related quality of life**^**3**^	***N*** = **152**	***N*** = **49**	***N*** = **103**			***N*** = **152**	***N*** = **96**	***N*** = **56**		
**EQ-5D-5L domain**										
Mobility	25 (16%)	0 (0%)	25 (24%)	<0.001	1	9 (5.9%)	1 (1.0%)	8 (14%)	0.001	12.23 (1.39-107.30)
Self-care	9 (5.9%)	0 (0%)	9 (8.7%)	0.03	1	3 (2.0%)	1 (1.0%)	2 (3.6%)	0.3	1.56 (0.13-19.11)
Usual activities	62 (41%)	3 (6.1%)	59 (57%)	<0.001	21.33 (6.15–74.00)	31 (20%)	6 (6.3%)	25 (45%)	<0.001	11.64 (4.18-32.46)
Pain or discomfort	87 (57%)	3 (6.1%)	84 (82%)	<0.001	67.60 (18.92–241.50)	39 (25%)	4 (4.2%)	34 (61%)	<0.001	38.21 (11.56-126.29)
Anxiety or depression	91 (60%)	17 (35%)	74 (72%)	<0.001	4.72 (2.26–9.83)	50 (33%)	18 (19%)	32 (57%)	<0.001	5.03 (2.32-10.88)
**EQ-5D-5L index**										
Median [IQR]	0.92 [0.80-0.97]	1.00[0.97–1.00]	0.87 [0.68–0.92]	<0.001		1.00[0.94–1.00]	1.00[1.00–1.00]	0.92 [0.87–0.96]	<0.001	
Mean (SD)	0.83 (0.21)	0.96 (0.09)	0.77 (0.23)			0.94 (0.12)	0.99 (0.04)	0.87 (0.17)		
**EQ-VAS**										
Median [IQR]	70 [50–80]	80 [75–91]	65 [49–75]	<0.001		80 [70–90]	85 [77–93]	75 [60–84]	<0.001	
Mean (SD)	66.6 (22.9)	82.3 (12.6)	59.2 (22.9)			78.4 (16.6)	83.3 (12.7)	70.0 (19.2)		

aOR=adjusted odds ratio, EQ-5D-5L=EuroQol 5-dimension, 5-level, EQ-VAS= EuroQol Visual Analogue Scale, IQR=interquartile range, SD=standard deviation,

^1^Logistic regressions were run separately for clinical progression and for each EQ-5D-5L domain with age and sex included as covariates in the model.

^2^Includes any individual who participated in any follow up (*N* = 221 at 3–6 months; *N* = 231 at 12–18 months).

^3^Only includes individuals who completed both EQ-5D-5L and EQ-VAS at both follow-up timepoints (*N* = 152).

The proportion of individuals requiring medication decreased from 92% (235/256) at discharge to 13% (31/231) at 12–18 months, which included 9% (20/231) on anti-inflammatories, 7% (16/231) on other cardiac medications including antihypertensives and beta-blockers, and none on anti-arrhythmic medication. Restrictions on exercise/physical activity decreased from 66% (168/256) at discharge (all clinician recommended) to 8% (19/231) at 12-18 months (all self-initiated). Hospital representation decreased from 27% (60/221) at 3–6 months to 20% (47/231) at 12–18 months; with 18% (41/231) to emergency department, 5.6% (13/231) hospital admission, and 1.3% (3/231) ICU admission at 12-18 months (Table 2). No individual required surgical intervention or additional procedures relating to their myocarditis diagnosis, during the follow up period. There were no deaths reported. Females were more likely to have hospital representations and remain on medications (Table 2).

After adjusting for age and sex, individuals with ongoing symptoms had higher odds of ongoing medication requirements, physical activity restrictions, and hospital representation at both 3-6 months (adjusted odds ratio aOR 3.52 (95% CI: 1.62–7.67), 6.13 (95% CI 2.84–13.24), 2.01 (95% CI 0.89-4.51 respectively) and 12–18 months (aOR 8.02 (95% CI: 2.62–24.55), 37.47 (95% CI: 4.48–313.18), 2.19 (95% CI: 0.98–4.90) respectively) compared to those without ongoing symptoms (Table 3).

Following clinical review and on clinician recommendation, 53 cases (22.9%) had another COVID-19 vaccination in the follow up period (median time to revaccination 166 days [IQR 121-207 days]); 19 Astrazeneca ChAdOx1-S (AZD1222), 8 Pfizer–BioNTech BNT162b2 and 26 Novavax NVX-CoV2373, all of whom were asymptomatic at the time of revaccination. Females were more likely to receive another COVID-19 vaccination (Table 2). Eight individuals reported self-resolving cardiorespiratory symptoms on revaccination (four following Astrazeneca ChAdOx1-S and four following Novavax NVX-CoV2373, all as their second COVID-19 vaccine dose). All sought medical review and investigation of myocarditis following subsequent vaccination, with no evidence or diagnosis of myocarditis recurrence found.

**Fig. 3:**
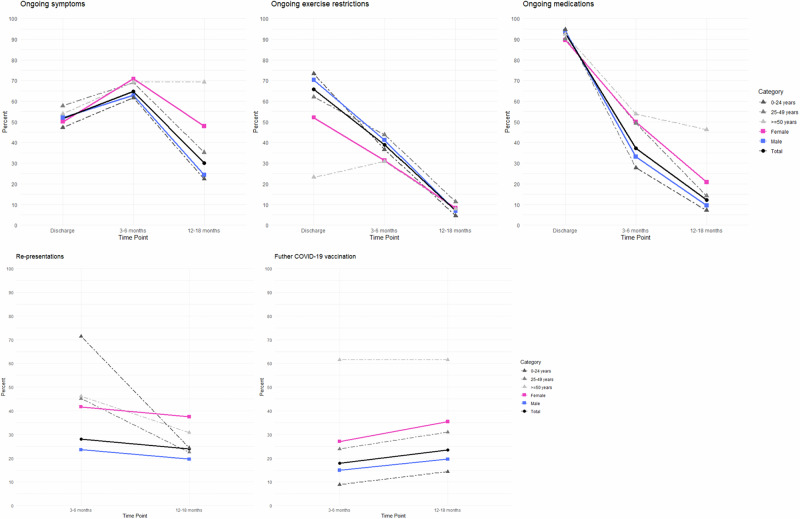
Progression of symptoms, exercise restrictions, medications, re-presentations and further COVID-19 vaccinations by sex and age, in myocarditis cases following mRNA COVID-19 vaccination who completed both 3-6 and 12-18 month follow up (*n* = 196).

As not all individuals completed follow-up at both timepoints, the progression of symptoms, exercise restrictions, medications, re-presentations, and further COVID-19 vaccinations by sex and age was examined in 196 (77%) cases who completed both follow-up timepoints (Fig. 2b, Fig. 3). In this subset, there was an increased proportion of individuals reporting symptoms at 3-6 months compared to discharge following initial diagnosis (Fig. 2b and Fig. 3), though the proportion reporting symptoms at 12-18 months was similar to that of the larger group (Fig. 2b vs Fig. 2a). Individuals in the older age groups were more likely to have ongoing symptoms and ongoing medication requirements. Those aged <24 years had the highest hospital re-presentation rates at 3-6 months, mostly to emergency departments only, but by 12-18 months, they had the lowest rate of ongoing symptoms, exercise restrictions, and medication requirements (Fig. 3).

### Quality of life

**Fig. 4:**
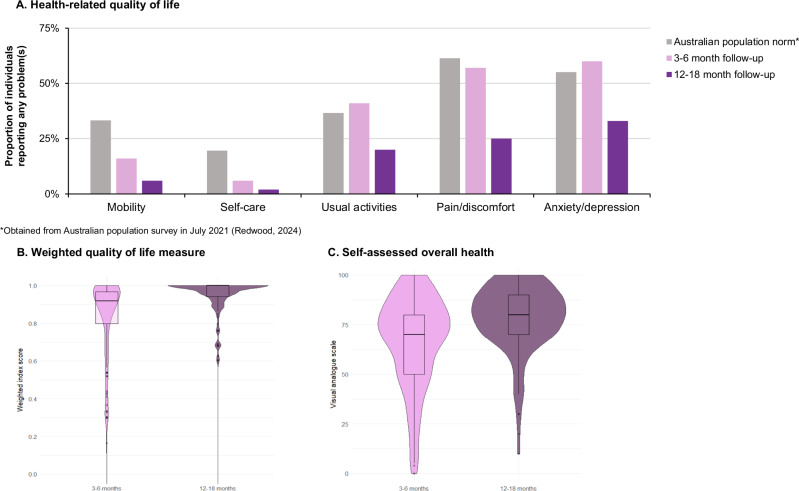
Self-assessment of health-related quality of life among myocarditis cases following mRNA COVID-19 vaccination who completed EuroQol 5-dimension, 5-level (EQ-5D-5L) questionnaire at both 3–6 and 12–18 month follow up (n = 152). **A** Health-related quality of life. **B** Weighted quality of life measure. **C** Self-assessed overall health.

EuroQol 5-dimension, 5-level (EQ-5D-5L) questionnaire and EuroQol Visual Analog Scale (EQ-VAS) was completed at both follow-up timepoints in 83% (152/183) of individuals aged 18 and over. In this subset of individuals, the proportion reporting problems decreased in all domains over time; from 16% at 3–6 months to 6% at 12–18 months for problems with mobility, from 6% to 2.0% for self-care, from 41% to 20% with performing usual activities, from 57% to 25% with pain/discomfort, and from 60% to 33% with feeling anxious or depressed (Fig. 4A). Of those who reported problems, most were slight to moderate in severity (Supplementary Fig. 1). As there was no baseline EQ-5D-5L data on cases prior to their diagnosis nor a control group, EQ-5D-5L data from the Australian population surveyed in July 2021^18^ is included for comparison (Fig. 4A).

Overall, individuals reported having good health which improved with time; EQ-5D index increased from 0.92 [IQR 0.80–0.97] at 3–6 months to 1.00 [0.94–1.00] at 12–18 months. However, individuals with ongoing symptoms had significantly lower EQ-5D-5L index scores and higher odds of reporting problems across all 5 domains at both timepoints after adjusting for age and gender (Table 3, Fig. 4B).

Overall, individuals reported having good health with a median overall health status (EQ-VAS) score of 70 [IQR 50–80] at 3–6 months and improving to 80 [IQR 70–90] at 12–18 months (Fig. 4B). Individuals reporting ongoing symptoms had significantly lower EQ-VAS compared to those without symptoms (65 [IQR 49–75] vs. 80 [IQR 75–91] at 3-6 months, 75 [IQR 60–84] vs 85 [IQR 77–93] at 12–18 months) (Table 3, Fig. 4C).

## Discussion

This is the longest prospective, comprehensive follow-up study of individuals diagnosed with myocarditis following mRNA COVID-19 vaccination to date that includes direct assessment of the individuals’ clinical progression and health-related quality of life. In this national study, we followed-up 256 individuals who had confirmed or probable myocarditis following receipt of an mRNA COVID-19 vaccine.

Over 18 months, there was overall clinical improvement with a significant reduction in the proportion of individuals with ongoing medication requirements (13%), exercise restrictions (8%), and hospitalisations (5.6%), no reports of further surgical or procedural intervention required and no deaths. Our findings are consistent with a recent population based study which reported similarly low rates of hospitalisation in the 18 months following post-vaccine myocarditis compared to myocarditis from other causes, confirming a milder clinical course^17^. It contrasts with the high rates of interventions including extracorporeal membrane oxygenation, ventricular assisted device, and heart transplantation reported following viral or acute myocarditis of other causes across the same timeframe^19–22^.

While medical care decreased, a proportion of individuals reported ongoing symptoms at 18 months. This finding builds on past studies^15,16^ that identified ongoing symptoms at 3–6 months and suggests a longer trajectory of illness for some individuals^11^. Determinants for ongoing symptoms at 12–18 months included female gender and older age, though it remains unclear why. Despite this, there was an increased frequency of both females and older individuals having further COVID-19 vaccinations, potentially because existing vaccination recommendations focus on the risk of vaccine-associated myocarditis in young males^23^. This highlights the need for further studies to understand the pathophysiology of vaccine-associated myocarditis to better characterise the clinical phenotype. This will not only assist in risk benefit-analyses and recommendations for future COVID-19 and other mRNA-based vaccines at both the individual and population level but also potentially for other vaccines where vaccine-associated myocarditis has been reported^24^.

Ongoing symptoms were also associated with significantly higher rates of ongoing exercise restriction, medication requirement, and hospital representation, though the association for hospital representation was much lower. This suggests the reported ongoing symptoms are clinically significant but not severe. Non-specific symptoms such as lethargy may also have been influenced by external factors, such as *SARS*-*CoV*-*2* infection and associated complications including long COVID, psychosocial impact of the pandemic, and the psychological distress of having a serious adverse event^25^, none of which were measured in this study.

Revaccination mostly occurred 6 months following diagnosis, all in asymptomatic individuals and more in females and older people. Reassuringly, none had myocarditis recurrence including 8/53 (15%) who received another mRNA vaccine, suggesting that revaccination upon symptom resolution can safely occur. This is consistent with findings that the risk of myocarditis following mRNA COVID-19 vaccines decrease with longer dosing intervals^26^, an important consideration for future vaccination recommendations. Establishment of an international revaccination registry assist would assist immensely in understanding the risk of AEFI recurrence at a population level.

In our health-related quality of life assessment, we found a higher proportion of individuals reported problems in all five domains on EQ-5D-5L with a corresponding lower median weighted index score and lower median overall health status (EQ-VAS) score at 3–6 months compared to Kracalik et al.’s study^15^. As Kracalik et al. only examined individuals aged 12–29 years, the difference in health-related quality of life may be related to more severe clinical picture in older individuals. However, all measures improved by 12-18 months to a level below those reported by Kracalik et al.^15^. While we did not have a control group, our cohort reported better health-related quality of life at 12–18 months compared to the Australian population surveyed in July 2021 across all five EQ-5D-5L domains (Fig. 4A), with higher estimated mean EQ-5D-5L index and EQ-VAS score (EQ-5D-5L index of 0.94 (0.12) vs 0.86 (SD 0.19) and EQ-VAS score of 78.38 (SD 16.64) vs 73.2 (SD 21.7))^18^. Individuals with ongoing symptoms, however, reported poorer health-related quality of life with lower estimated mean EQ-5D-5L index and EQ-VAS score compared to those without ongoing symptoms. Reassuringly, their scores were similar compared to the population norm, affirming that the impact of vaccine-associated myocarditis on quality of life, even in those with ongoing symptoms, are mild.

The strength of this study lies in the comprehensive case ascertainment through established links between researchers and the national spontaneous vaccine safety surveillance system. Our cases were all clinically verified and classified against internationally accepted case definitions.

Access to medical records enabled the collection of detailed clinical data. Direct follow-up with the individuals enabled characterisation of symptoms, recording clinical care beyond hospitalisations, and health-related quality of life that otherwise cannot be obtained from studies relying on health datasets alone.

This study has some limitations. Firstly, it is possible that not all cases of myocarditis in Australia associated with mRNA COVID-19 vaccination were reported to the TGA’s spontaneous (passive) adverse event reporting system and therefore captured in this analysis, particularly the milder cases. Secondly, only half of all confirmed and probable cases reported to the TGA consented to the follow-up study. The consenting cohort was older, had a more severe initial clinical presentation and significantly higher rate of ongoing symptoms at discharge compared to those not followed-up. This indicates a selection bias towards a more clinically serious or phenotypically different cohort that may not be generalisable to all post-vaccination myocarditis cases. Responder bias may also explain the higher rate of ongoing symptoms at 3–6 month follow up compared to time of discharge, further impacting the generalisability of this study’s findings. Correlation with population-based studies utilising linked health datasets may assist in providing more objective measures of subsequent healthcare requirements following diagnosis and in contextualising clinical outcomes in this follow-up cohort compared to the wider population. Thirdly, we had no control group for comparison. Ongoing symptoms of chest pain and lethargy and its impact on health-related quality of life may be multifactorial and not directly related to the individuals’ myocarditis diagnosis. Finally, past studies have found late gadolinium enhancement indicates irreversible myocardial damage and predicts cardiac and all-cause mortality^6,15,27–32^. We could not correlate our clinical findings with cMRI results, as only 27% of the follow-up cohort had a cMRI at diagnosis and thus it is not possible to identify imaging markers of longer-term sequalae in our cohort.

Most individuals with myocarditis following mRNA COVID-19 vaccination showed significant clinical and health-related quality of life improvement in the first 12–18 months following diagnosis. While there was a proportion of individuals who reported ongoing symptoms with associated risk of ongoing medication requirements, exercise restrictions, and poorer self-reported health-related quality of life, clinical severity remained mild, with low hospitalisation rates and no deaths. These findings support ongoing use of mRNA COVID-19 vaccines in at risk individuals to prevent disease caused by *SARS*-*CoV*-*2* infection, which is associated with a greater risk of myocarditis compared to vaccination^33,34^. Follow up and further research into the aetiology of those with ongoing symptoms using a biopsychosocial model is critical in the management of these individuals.

## Methods

### Study design and population

AEFIs are reported to Australia’s national regulatory agency, the TGA, by healthcare providers, individuals, state/territory health departments, and pharmaceutical companies. Reporting of serious AEFI to state/territory health departments by healthcare providers is mandated in some states and territories in Australia and was actively undertaken in others during the COVID-19 vaccine rollout.

Reporters of possible myocarditis cases were contacted by pharmacovigilance staff at the TGA for missing and/or additional information to enable case confirmation and classification. Cases reported from the commencement of mRNA COVID-19 vaccine use in Australia in February 2021 to 5 July 2022 and classified by TGA staff as confirmed or probable myocarditis following a monovalent mRNA COVID-19 vaccine using the modified U.S. CDC case definition (Supplementary Table 1)^14^ were included in this study (Fig. 1). During this period, Pfizer–BioNTech BNT162b2 and Moderna mRNA-1273 were used for primary doses for individuals aged 5 years and older with a recommended minimum interval of 3 weeks between first and second primary doses^35^. Individuals were contacted by a study investigator (state/territory health department or research staff member) and invited to participate in follow-up.

### Data collection

Following case identification by the TGA, demographic and clinical data were collated from medical records by study investigators under a waiver of consent. Individual demographics, medical history and potential cardiovascular risk factors, presenting clinical features, laboratory results (serum troponin), ECG, echocardiographic and cMRI findings, pathology reports, acute and long-term treatment, hospital management, ICU admission, surgical intervention, clinician-recommended exercise/physical activity restriction, and discharge outcomes following initial presentation were collected. Vaccination date (and time to onset of symptoms) and vaccine brand was confirmed by the Australian Immunisation Register.

Individuals who were diagnosed with myocarditis following COVID-19 vaccination were reviewed by cardiologists, following the general management approach for viral myocarditis. An individual’s first follow-up with a cardiologist was usually 3-6 months following diagnosis with further investigations and frequency of follow-up determined by the clinician based on clinical severity, local practices and access to specialist care and facilities. Due to this variability, data collection was focused on patient reports and hospital records.

Informed consent to participate in the follow-up was sought from cases (or their parent or legal guardian in the case of children under the age of 16). For those who consented to follow-up, data on their clinical progression following diagnosis was collected by research staff using standardised case report forms at 3-6 months (following their initial cardiologist follow-up) and 12-18 months following diagnosis. Clinical data including new/persistent (hereafter ongoing) symptoms, clinical management, complications, ongoing exercise restrictions (either medically recommended or self-initiated), whether they returned to baseline health, and subsequent COVID-19 vaccination(s) and associated outcome(s) were collected through phone interviews with individuals or their parent/guardian at each time point. For individuals aged 18 or older, self-reported health related quality of life following myocarditis diagnosis was assessed using the standardised EQ-5D-5L questionnaire across five dimensions: mobility, self-care, performing usual activities, pain or discomfort, and anxiety or depression with five severity levels from no problems to extreme problems, and then categorised into no problems (severity level 1) or any problems (severity levels 2–5)^36^. Responses were converted to an EQ-5D summary index value between 0 (representing death) and 1 (representing perfect health) for each individual using a weighted scoring algorithm based on Australian population data^18^. Individuals provided a self-rating for overall health using EQ-VAS, with scores ranging from 0 (worst possible health) to 100 (best possible health).

### Statistical analysis

Categorical variables are described as numbers and percentages or as medians, ranges, and interquartile range (IQR). Continuous variables are described by median and IQR for non-normally distributed data. Variables were compared using chi-squared or Fisher’s exact test for categorical values and Mann-Whitney U for non-parametric continuous variables where appropriate. A logistic regression model was used to investigate the association between ongoing symptoms and care progression (ongoing medications, exercise restrictions and hospital representations); and between ongoing symptoms and quality of life (dichotomised EQ-5D-5L responses). Analyses were conducted in R version 4.1.1.

## Supplementary information

Supplementary material

## Data Availability

The datasets generated and analysed during the current study are not publicly available to protect the sensitive information of individuals who participated in the study that may be potentially identifiable but are available from the corresponding author on reasonable request.

## Code availability

The underlying code for this study is not publicly available but may be made available to qualified researchers on reasonable request from the corresponding author.
