# Supplementary material for: Surveillance and follow up outcomes of myocarditis after mRNA COVID-19 vaccination in Australia
Source: NPJ Vaccines. 2025 Jul 16;10:155. doi: 10.1038/s41541-025-01206-w (PMC12267834; doi:10.1038/s41541-025-01206-w)
Supplement: Supplementary file 1 — Supplementary material [file 41541_2025_1206_MOESM1_ESM.docx]

**Supplementary Table 1. Case definition criteria for myocarditis following mRNA COVID-19 vaccination based on a US Centers for Disease Control and Prevention (CDC) case definition**

| **Case status** |  | **Criteria^1^** |
| --- | --- | --- |
| Confirmed |  | Presence of ≥1 new or worsening of the following clinical symptoms:   - Chest pain, pressure, or discomfort - Dyspnoea, shortness of breath or pain with breathing - Palpitations - Syncope   AND  ≥1 new finding of   - Histopathologic confirmation of myocarditis - cMRI findings consistent with myocarditis in the presence of troponin level above upper limit of normal (any type of troponin)   AND   - No other identifiable cause of symptoms and findings |
| Probable |  | Presence of ≥1 new or worsening of the following clinical symptoms:   - Chest pain, pressure, or discomfort - Dyspnoea, shortness of breath or pain with breathing - Palpitations - Syncope   AND  ≥1 new finding of   - Troponin level above upper limit of normal (any type of troponin) - Abnormal ECG^2^ or rhythm monitoring findings consistent with myocarditis - Abnormal cardiac function or wall motion abnormalities on echocardiogram - cMRI findings consistent with myocarditis   AND   - No other identifiable cause of symptoms and findings |

cMRI=cardiac magnetic resonance imaging, ECG=electrocardiogram

1. Criteria is a modified version of the US Centers for Disease Control and Prevention (CDC) case definition, removing the symptoms in infants and children aged < 12 years (irritability, vomiting poor feeding, tachypnoea and lethargy) from the clinical symptom criteria

2. ECG findings include least one of the following: ST segment or T-wave abnormality; Paroxysmal or sustained atrial, supraventricular or ventricular arrhythmias; AV nodal conduction delays or intraventricular conduction defects

**Supplementary Table 2. Demographics, past medical history, clinical and diagnostic findings of initial presentation in 552 confirmed and probable myocarditis cases following mRNA COVID-19 vaccination presenting between 21 April 2021 and 5 July 2022**

|  | **All cases**  **N=552** | **Confirmed case**  **N=68** | **Probable Case**  **N=484** | ***P*** |
| --- | --- | --- | --- | --- |
| **Consented for follow up** | 256 (46%) | 34 (50%) | 222 (46%) | 0.5 |
| **Vaccine brand** |  |  |  |  |
| Pfizer–BioNTech BNT162b2 | 487 (88%) | 63 (93%) | 424 (88%) | 0.2 |
| Moderna mRNA-1273 | 65 (12%) | 5 (7.4%) | 60 (12%) |  |
| **Vaccine dose** |  |  |  |  |
| Dose 1 | 111 (20%) | 13 (19%) | 98 (20%) | 0.8 |
| Dose 2 | 389 (70%) | 47 (69%) | 342 (71%) |  |
| Dose 3 | 52 (9.4%) | 8 (12%) | 44 (9.1%) |  |
| **Age (years)** |  |  |  |  |
| Median [IQR] | 22 [17-33] | 22 (19, 34) | 21 (16, 33) | 0.13 |
| 0-11 | 6 (1.1%) | 1 (1.5%) | 5 (1.0%) |  |
| 12-14 | 70 (13%) | 0 (0%) | 70 (14%) |  |
| 15-19 | 165 (30%) | 25 (37%) | 140 (29%) |  |
| 20-24 | 90 (16%) | 11 (16%) | 79 (16%) |  |
| 25-29 | 49 (8.9%) | 11 (16%) | 38 (7.9%) |  |
| 30-39 | 145 (26%) | 17 (25%) | 128 (26%) |  |
| ≥40 | 27 (4.9%) | 3 | 24 |  |
| **Sex** |  |  |  |  |
| Male | 418 (76%) | 54 (79%) | 364 (75%) | 0.7 |
| Female | 132 (24%) | 14 (21%) | 118 (24%) |  |
| Another term | 2 (0.4%) | 0 (0%) | 2 (0.4%) |  |
| **Aboriginal and/or Torres Strait Islander** | 16 (2.9%) | 2 (2.9%) | 14 (2.9%) | >0.9 |
| **Past medical history** |  |  |  |  |
| Cardiovascular condition^1^ | 24 (4.3%) | 7 (10%) | 17 (3.5%) | 0.02 |
| Myocarditis | 9 (1.6%) | 4 (5.9%) | 5 (1.0%) | 0.02 |
| Pericarditis | 7 (1.3%) | 1 (1.5%) | 6 (1.2%) | 0.6 |
| Arrythmia | 6 (1.1%) | 0 (0%) | 6 (1.2%) | >0.9 |
| Congenital heart disease | 4 (0.7%) | 2 (2.9%) | 2 (0.4%) | 0.08 |
| Autoimmune/systemic inflammatory conditions^2^ | 16 (3.3%) | 16 (3.3%) | 16 (3.3%) | 0.7 |
| Genetic/chromosomal condition | 10 (1.8%) | 1 (1.5%) | 9 (1.9%) | 0.8 |
| **Family history of cardiac conditions^3^** | 29 (5.3%) | 3 (4.4%) | 26 (5.4%) | >0.9 |
| **Time to symptom onset** |  |  |  |  |
| 0-7 days | 465 (84%) | 55 (81%) | 410 (85%) | 0.5 |
| 8-10 days | 22 (4.0%) | 4 (5.9%) | 18 (3.7%) |  |
| ≥ 11 days | 65 (12%) | 9 (13%) | 56 (12%) |  |
| **Presenting symptoms** |  |  |  |  |
| Cardiovascular symptoms |  |  |  |  |
| Chest pain, pressure or discomfort | 530 (96%) | 62 (91%) | 468 (97%) | 0.04 |
| Heart fluttering, racing | 122 (22%) | 15 (22%) | 107 (22%) | >0.9 |
| Diaphoresis | 52 (9.4%) | 7 (10%) | 45 (9.3%) | 0.8 |
| Syncope | 12 (2.2%) | 4 (5.9%) | 8 (1.7%) | 0.048 |
| Dizziness | 49 (8.9%) | 9 (13%) | 40 (8.3%) | 0.2 |
| Respiratory symptoms |  |  |  |  |
| Shortness of breath (after exercise, at rest or lying down) | 190 (34%) | 27 (40%) | 163 (34%) | 0.3 |
| Cough | 31 (5.6%) | 3 (4.4%) | 28 (5.8%) | >0.9 |
| Pleuritic chest pain (pain on breathing) | 79 (14%) | 19 (28%) | 60 (12%) | 0.001 |
| Gastrointestinal |  |  |  |  |
| Abdominal pain | 11 (2.0%) | 3 (4.4%) | 8 (1.7%) | 0.14 |
| Nausea/vomiting | 70 (13%) | 13 (19%) | 57 (12%) | 0.09 |
| Diarrhoea | 5 (0.9%) | 0 (0%) | 5 (1.0%) | >0.9 |
| Loss of appetite | 7 (1.3%) | 0 (0%) | 7 (1.4%) | >0.9 |
| Systemic |  |  |  |  |
| Fever | 73 (13%) | 15 (22%) | 58 (12%) | 0.02 |
| Headache | 57 (10%) | 6 (8.8%) | 51 (11%) | 0.7 |
| Lethargy | 103 (19%) | 16 (24%) | 87 (18%) | 0.3 |
| **Investigations** |  |  |  |  |
| Abnormal ECG | 354/522 (64%) | 53/59 (90%) | 301/463 (65%) |  |
| Abnormal echocardiogram | 180/487 (33%) | 33/59 (56%) | 147/428 (34%) |  |
| Abnormal cMRI | 128/149 (86%) | 67/67 (100%) | 61/82 (74%) |  |
| Elevated troponin | 524/552 (95%) | 62/68 (91%) | 462/484 (95%) |  |
| Abnormal biopsy | 2/3 (67%) | 2/3 (67%) | 0 (0%) |  |
| **Management details** |  |  |  |  |
| Treated at GP | 15 (2.7%) | 2 (2.9%) | 13 (2.7%) | 0.07 |
| Treated in ED | 74 (14%) | 5 (7%) | 69 (14%) |  |
| Hospitalised, no ICU admission | 443 (82%) | 56 (82%) | 387 (80%) |  |
| Hospitalised, with ICU admission | 20 (3.7%) | 5 (7%) | 15 (3%) |  |
| **Intervention** |  |  |  |  |
| Non-invasive cardiorespiratory support | 10 (1.8%) | 4 (5.9%) | 6 (1.2%) | 0.007 |
| Intubation and ventilation | 2 (0.4%) | 1 (1.5%) | 1 (0.2%) | 0.2 |
| ECMO | 1 (0.2%) | 1 (1.5%) | 0 (0%) | 0.12 |
| Procedures/surgery | 12 (3.0%) | 1 (1.8%) | 11 (3.2%) | >0.9 |
| **Discharge medications** |  |  |  |  |
| Anti-inflammatories^4^ | 485 (88%) | 59 (87%) | 426 (88%) | 0.8 |
| Corticosteroids^5^ | 18 (3.3%) | 6 (8.8%) | 12 (2.5%) | 0.02 |
| Anti-arrhythmic | 1 (0.2%) | 0 (0%) | 1 (0.2%) | >0.9 |
| Anticoagulation | 33 (6%) | 3 (4.4%) | 30 (6.2%) | 0.8 |
| Diuretics | 3 (0.5%) | 2 (2.9%) | 1 (0.2%) | 0.04 |
| Other cardiac medications^6^ | 81 (15%) | 23 (34%) | 58 (12%) | <0.001 |
| **Discharge outcomes** |  |  |  |  |
| Ongoing symptoms | 147 (27%) | 20 (34%) | 127 (41%) | 0.3 |
| Placed on restrictions on physical activity | 291 (53%) | 46 | 245 | <0.001 |

*****Case with follow up includes those who consented to the study and responded to the 3-6 month follow-up and/or 12-18 month follow-up

cMRI=cardiac magnetic resonance imaging, ECG=electrocardiogram, ECMO=extracorporeal membrane oxygenation, ED=emergency department, GP=general practice, ICU=intensive care unit

1. Cardiovascular condition includes myocarditis, pericarditis, arrhythmia, congenital heart disease

2. Autoimmune/systemic inflammatory conditions include rheumatoid arthritis, scleroderma, systemic lupus erythematosus, Sjogren’s syndrome, Kawasaki disease, inflammatory bowel disease

3. Family history includes sudden cardiac death, cardiomyopathy, permanent pacemaker, implantable cardioverter defibrillator device

4. Anti-inflammatory medications include aspirin, ibuprofen, naproxen and colchicine

5. Corticosteroids include prednisone, dexamethasone and methylprednisone

6. Other cardiac medications include ACE inhibitors, angiotensin receptor blockers and beta blocker

**Supplementary Figure 1. Self-assessment of health-related quality of life by level of severity on EuroQol 5-dimension, 5-level (EQ-5D-5L) questionnaire among myocarditis cases following mRNA COVID-19 vaccination at 3-6 and 12-18 months (n=152)**

**
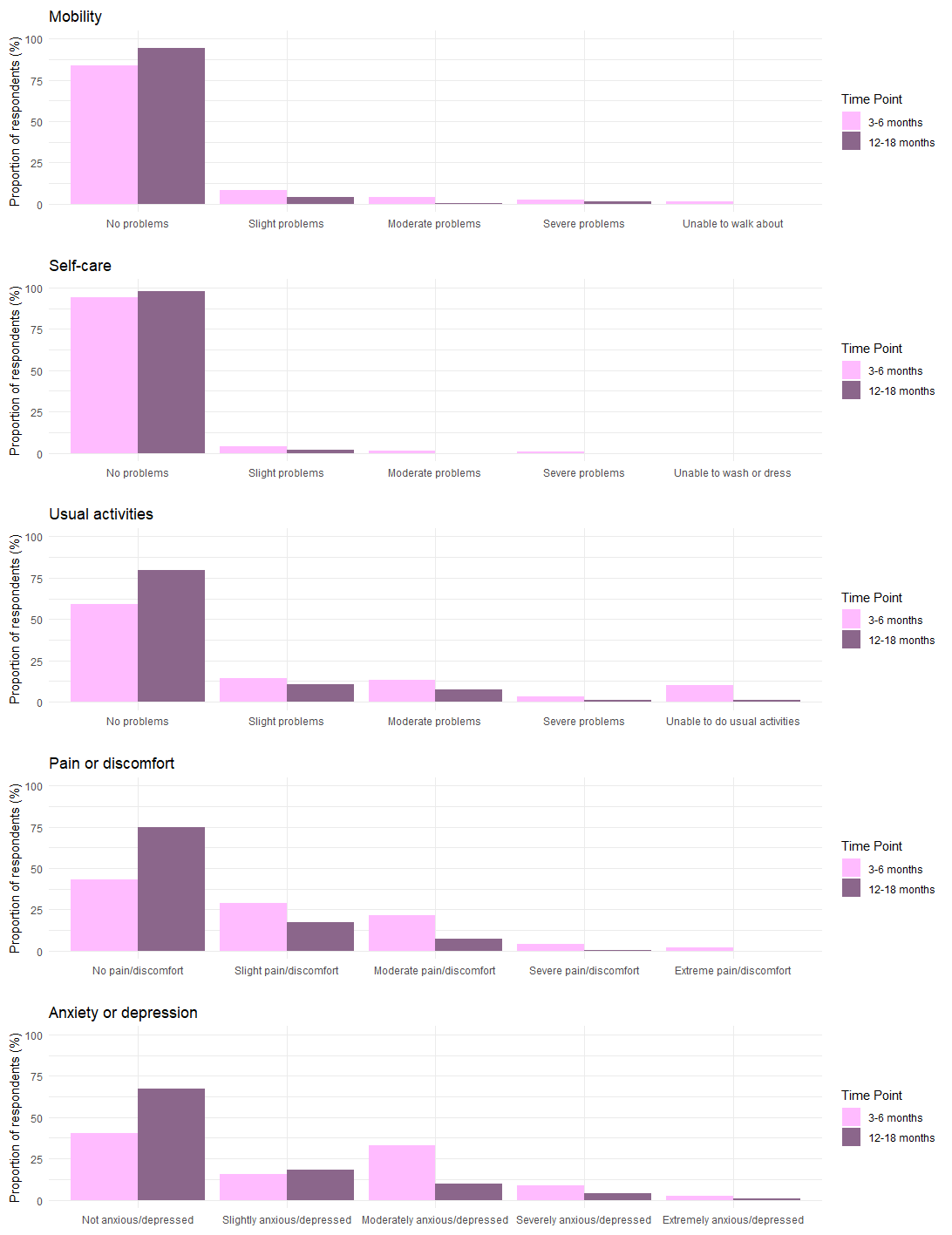
**
